# Gender harmony: improved standards to support affirmative care of gender-marginalized people through inclusive gender and sex representation

**DOI:** 10.1093/jamia/ocab196

**Published:** 2021-10-06

**Authors:** Robert C McClure, Caroline L Macumber, Clair Kronk, Chris Grasso, Robert J Horn, Roz Queen, Steven Posnack, Kelly Davison

**Affiliations:** 1 MD Partners, Inc., Lafayette, Colorado, USA; 2 Clinical Architecture, Carmel, Indiana, USA; 3 Department of Biomedical Informatics, University of Cincinnati College of Medicine, Cincinnati, Ohio, USA; 4 Fenway Health, Boston, Massachusetts, USA; 5 Fairhaven Technology, Maynard, Massachusetts, USA; 6 School of Health Information Science, University of Victoria, Victoria, BC, Canada; 7 US Department of Health and Human Services, Office of the National Coordinator for Health IT, Washington, District of Columbia, USA; 8 Canada Health Infoway, Toronto, ON, Canada; 9 School of Health Information Science, University of Victoria, Victoria, BC, Canada

**Keywords:** inclusive design, gender-marginalized people, affirmative care, health information standards, gender harmony model

## Abstract

**Objective:**

Accurate representation of clinical sex and gender identity in interoperable clinical systems is a major challenge for organizations intent on improving outcomes for sex- and gender-marginalized people. Improved data collection has been hindered by the historical approach that presumed a single, often binary, datum was sufficient. We describe the Health Level Seven International (HL7) Gender Harmony logical model that proposes an improved approach.

**Materials and Methods:**

The proposed solution was developed via an American National Standards Institute (ANSI)-certified collaborative balloted process. As an HL7 Informative Document, it is an HL7 International-balloted consensus on the subject of representing sex and representing gender in clinical systems based on work of the gender harmony project led by the HL7 Vocabulary Work Group.

**Results:**

The Gender Harmony Model is a logical model that provides a standardized approach that is both backwards-compatible and an improvement to the meaningful capture of gender identity, recorded sex or recorded gender, a sex for clinical use, the name to use, and pronouns that are affirmative and inclusive of gender-marginalized people.

**Conclusion:**

Most clinical systems and current standards in health care do not meaningfully address, nor do they consistently represent, sex and gender diversity, which has impeded interoperability and led to suboptimal health care. The Gender Harmony Project was formed to create more inclusive health information exchange standards to enable a safer, higher-quality, and embracing healthcare experience. The Gender Harmony Model provides the informative guidance for standards developers to implement a more thorough technical design that improves the narrow binary design used in many legacy clinical systems.

## BACKGROUND 

It has been over 7 years since the *Transgender Tipping Point* (title of a *Times* magazine article published on May 29, 2014, used to describe a shift in increased visibility of transgender people in popular culture)^1^ and, in some ways, the healthcare system has only gotten more hostile toward transgender persons^2–6^ . Thirty-three percentage (33%) of transgender persons in the United States have had at least one negative experience with a healthcare provider and 23% have reported not seeking care when needed due to fear of mistreatment.^7^ Some societies in the world still require forced sterilization of transgender people, and others stigmatize them with mental health diagnoses rather than challenging outdated norms and creating inclusive spaces.^8–11^ In India and Nepal, considerations for a third gender have been added to the national census, but without input from the affected communities.^12–14^ It was not until 2019 that *hijra* (an officially recognized third gender in the Indian subcontinent who are considered neither completely male nor female) could even vote in Bangladesh.^15^ In the United Kingdom, waitlists for providers experienced in providing gender-affirming care are several *years* long because there are so very few available.^16–19^ And yet the National Health Service has recently attempted to dismantle protections and treatments for transgender youth.^20^ Social stigma and barriers to access have led to disproportionate mortality of transgender people from COVID-19 and inappropriate medical care.^18^^,^^21–26^ Intersex (individuals born with any of several sex characteristics that do not fit typical binary notions of male or female bodies) patients live with a greater burden of data-related harms, suffering sexual and emotional abuse, gaslighting, obfuscation of clinical records, forced sterilization, and unnecessary surgeries, often to fit within the Eurocentric sociocultural binary normativism.^8^^,^^11^^,^^27–30^ It is important to understand that intersex patients experience clinical and life situations quite distinct from transgender individuals and represent unique challenges for clinical documentation and affirming care.

We have known for 2 decades that implementing inclusive sex and gender data collection practices in clinical systems is a critical first step to eliminating data invisibility and addressing health disparities for gender-marginalized people,^31–33^ but accurate representation of sex and gender diversity in clinical systems and standards is a challenge for many reasons, and adoption has been slow and sporadic. Cisheteronormative (the concept that being cisgender [nontransgender] and heterosexual is a preferred, default, or otherwise “normal” aspect of society and that anything outside of those strict boundaries is “deviant” or “abnormal”) bias in society and in health care is leading to preventable harms for gender-marginalized people.^34^ Gender-affirmative care includes the appropriate use of person-centered language such as used name(s), pronoun(s), and possessive adjectives^35^ and provides objective and appropriate health screening, treatment, and referral options based on a person’s needs and clinical sex characteristics. Gender-affirming agencies provide welcoming care environments with gender-neutral bathroom options and inclusive milieu and that otherwise celebrate diversity and inclusivity while avoiding harmful healthcare practices of misnaming (to refer to a person using the wrong name or names, either accidentally or deliberately, in situations where such names are inappropriate or harmful; usually used in reference to people using transgender persons’ dead names [ie, names utilized by transgender persons before transitioning or coming out]), misgendering (to refer to a person using terms that express the wrong gender, either accidentally or deliberately, in situations where such expressions are inappropriate or harmful; for example, calling a woman “son” or a man “girl”), and outing people (the deliberate or accidental disclosure of an individual’s status as 2SLGBTQIA+ [acronym meaning two-spirit, lesbian, gay, bisexual, transgender, queer, intersexual, asexual, while “+” indicates that some individuals may express gender and sexuality in others ways], without their consent).^36^^,^^37^ Please see Table 1 for additional key terms.^38^Culturally safe care is provided when the patient voice is central to care, and when the behaviors, attitudes, policies, and practices of an organization support safe interactions between patients and providers.^39^ Practices that support inclusive sex and gender healthcare information capture and exchange can support culturally safe and gender-affirming health care and theoretically reduce the effects of stigma on health outcomes.^36^^,^^40^

**Table 1.** Glossary of additional key terms

Glossary of additional key terms	
Term	Definition
Administrative gender	A phrase found in some clinical systems and health information standards wherein the value is intended to support determination of “administrative” activities, such as bed assignment. It is presumed that as an “administrative” value, this “gender” may not be reflective of the patient gender in all contexts.
Administrative sex	Like administrative gender, this phrase is found in some clinical systems and health information standards wherein the value is intended to support determination of “administrative” activities, when those activities are expected to be aligned with “sex” characteristics. The distinction between this phrase and administrative gender is unclear in most implementations and cannot be reliably distinguished from other “administrative gender.”
Gender expression	How a person chooses to outwardly express their gender, including behavior, speech, clothing, names, and pronouns used. Gender expression is context-dependent. People may not feel safe expressing their felt gender in certain spaces or with certain people because of the risk of discrimination.
Gender identity (GI)	Personal identification with a gender term such as *man*, *woman*, *nonbinary*, *transgender*, and *Two-Spirit.* A person may have a gender *identity or identities.* Gender identity cannot be an externally applied label. It is something that is shared when a person feels safe to share it. Cisgender people generally have a binary gender identity that matches their sex assigned at birth. A transgender person’s gender identity typically does not. People whose gender identity does not match their sex at birth may or may not identify as transgender; they may identify with the binary gender term that matches their gender identity, or they may also identify as nonbinary.
Name used	The name a person wishes to use, which may be different than their legal name.
Nonbinary	Describes a person whose gender identity falls outside of the traditional gender binary structure of girl/woman and boy/man.
Pronouns	The pronouns (eg, him/her/they/ze) and possessive adjectives (eg, his/hers/theirs) a person wishes to be addressed by.
Sex	The concept of sex is a biological construct and pertains to a person’s genetics, hormones, and anatomy. Sex is most often represented by the terms *male*, *female*, and *intersex*, which is assigned at birth Intersex people may be assigned a sex of *male* or *female* at birth. For example, persons with nonmosaic Klinefelter’s are typically sexed male, while persons with nonmosaic Turner’s are typically sexed female.
Sex for clinical use (SFCU)	A newly proposed sex characteristic defined within the HL7 gender harmony model. Used to represent a clinical sex value for use when considering a specific clinical observation or activity.
Transition	The term *transition* in this context is a highly variable and deeply personal process by which social, behavioral, and sometimes biological sex and gender identity characteristics are aligned with a person’s felt gender.
Two-spirit	The term *Two-Spirit* was created by Indigenous people for use by Indigenous people, and may be used as an Indigenous gender, sexual, or spiritual identity.

The objective of this paper is to outline the work of the Gender Harmony Project (GHP) which has, since 2019, developed a gender-inclusive Health Level Seven International (HL7) logical model: the HL7 Gender Harmony Model (the Model).^41^^,^^42^ Within our model, sex is used to classify individuals as female, male, or specified (neither female nor male) and can be based on an infant’s anatomy, other biological characteristics, or can be associated with physical and physiological features.^42^^,^^43^ Gender is defined as a person’s inner sense of being a girl/woman/female/feminine, boy/man/male/masculine, nonbinary (Both “nonbinary” and “non-binary” spellings are used in the community.), something else, or having no gender.^42^^,^^43^ Gender identity (GI) can also be referred to as simply a person’s “gender.” Fundamentally, the Model is about providing clinicians the information required to support informed and safe health care for every patient based on accurate representation of gender and sex without undue workflow changes.

In the next sections of this document, we will outline the primary challenges of the GHP, provide a rationale for change, and present the current state of messaging standards in relation to sex and gender data elements. We will end the paper by discussing the Model, the method used by the GHP to generate the Model and will then discuss current efforts of standards development organizations (SDOs) to set the conditions for inclusive and affirming health care. The scope of the Model is focused on the improved representation of (clinical) sex and gender identity, along with supporting data elements, and does not address sexual orientation or behavior. Our primary focus is on people who are marginalized in health care because of their sex and or gender, including people who are intersex, transgender, nonbinary, and Two-Spirit. Terminological standards, which include reference terminologies such as SNOMED CT, are mentioned but not addressed in detail. The set of suggested baseline values to be reported for each of the model elements is provided in Supplementary Appendix S1. In addition, an acronym list is provided in Supplementary Appendix S2.

## PROBLEM STATEMENT

The current representation of patient sex and gender information in interoperable clinical systems poses major challenges for organizations intent on improving outcomes for sex- and gender-marginalized people. Such challenges include:

- lack of common understanding of sex and gender terminology,^36^^,^^40^
- conflation of administrative and clinical sex and gender coding in clinical systems,^44^
- binary representation of sex and gender (ie, male/female and man/woman),^36^^,^^40^^,^^44^
- use of “other” and “undifferentiated” values to represent diversity,^36^^,^^40^^,^^44^
- the assumption that GI is static,^36^ and
- the presumption that quality clinical care can be delivered for all individuals based solely on administrative sex or administrative gender.

This is by no means a complete representation of the challenges in this complex domain but should be sufficient for most readers to understand both the problems with current sex and gender representation and the resulting rationale for the different elements of the Model.

### Sex and gender in clinical systems

An important consideration when discussing sex and gender in the context of health information systems standards is that gender (occasionally labeled as “sex”) is often initially captured for administrative and legal tasks. These representations are not always suitable for clinical care and are often exchanged with clinical data elements as though they are synonymous.^44^ Recognition that the current datums of administrative sex and administrative gender are not synonymous or interchangeable for the GPM elements of sex for clinical use (SFCU - defined below) and gender identity (GI) is essential to enabling affirming clinical interactions and equitable patient outcomes and to changing when, how, and where these data are recorded, exchanged, and used.^36^^,^^40^^,^^44–49^

#### Binary representation and conflation of terms

The vast majority of data elements used to capture sex and gender information use codes and attributes that enforce an oversimplified and deeply entrenched binary construct of sex and gender (ie, “man”/“woman,” “male”/“female,” and “M”/“F”) that privilege cisgender people and exclude nonbinary, transgender, gender nonconforming, Two-Spirit, and intersex people.^44^ Many systems and standards provide only a single field to capture disparate sex and gender data such as “Birth Sex,” “Administrative Gender,” or simply “Sex.”^36^^,^^40^^,^^44^ This structural bias leads directly to the invisibility of sex- and gender-marginalized people in health data and all but omits their data from improvement analytics, clinical decision support, research, and their associated benefits.^40^^,^^50^^,^^51^ Systems-induced invisibility also deprives clinicians of valuable information that can support gender-affirming clinical interactions and safe decisions leading to higher-quality clinical care.^40^^,^^39^^,^^51^

#### Gender identity may be fluid and context-dependent

The patient-driven, dynamic, and context-dependent nature of a person’s gender identity is not supported in systems that use single, static, permanent gender values. The assumption that gender is static and accurately represented by binary values for clinical interactions leads to nonaffirming healthcare interactions and avoidable harms.^36^

## CURRENT STATE OF SEX AND GENDER REPRESENTATION

While data capture and representation are almost always under the control of the specific application in use, exchange standards, and requirements defined by standards bodies and governmental agencies frequently have a strong influence on how a particular application captures and represents this information. An understanding of current approaches to sex and gender representation in standards provides important context for the specification and implementation of the Model. The current state of sex and gender element representation in various exchange standards provided by SDOs, along with a specification pertinent to interoperable health information exchange in the United States, is discussed in this section.

### Health Level 7 Version 2 

HL7^53^ is a family of widely adopted healthcare messaging standards that allow clinical systems to exchange information with one another via “messages” that contain clinical, demographic, visit, and provider information. The Version 2 (HL7 V2.x)^52^ messaging standard primarily supports administrative and laboratory exchange. HL7 V2 Admit Transfer Discharge (ADT) messages are the most widely used standards-based method. Early implementations of HL7 V2 had a single field (Patient Identifier, Segment 8 [PID-8]) named “Sex” with user-defined values. PID-8 is used in every HL7 V2 ADT message. From Version 2.4 forward, the field was renamed to “Administrative Sex” in recognition that it was insufficient or inappropriate for conveying sex information for clinical use. Even so, many systems still assume that PID-8 is the only representation of sex characteristics needed. There is an assumption that a single, permanent patient value can be prudently applied to care in all situations. The addition of other segments closed some implementation gaps by allowing a higher level of granularity to communicate administrative sex values for various actors (“Patient Administrative Sex,” “Insured’s Administrative Sex,” “Guarantor’s Administrative Sex,” “Next of Kin/Associated Party’s Administrative Sex,” etc.), but V2 still lacks differentiation between gender, sex, and SFCU.

### HL7 Version 3 and Consolidated Clinical Document Architecture 

The evolution from HL7 Version 2 to Version 3 (V3) product families allowed the new V3 standards, such as Consolidated Clinical Document Architecture (HL7 C-CDA),^54^ to be derived from a common core Reference Information Model (RIM) expressed using XML. In the V3 model, the “Administrative Sex” segment was acknowledged to be “Administrative Gender” and defined as “…the behavioral, cultural, or psychological traits typically associated with one sex.”^54^ The focus of this field remained nonclinical and is defined as “a high-level classification […] for the appropriate allocation of inpatient bed assignment.”^54^ The transcription of message information from V2 to V3 implementations, primarily in Clinical Documentation Architecture (CDA), has its origins in PID-8 Administrative Sex, with all of its ambiguity.

### HL7 Fast Healthcare Interoperability Resources 

Fast Healthcare Interoperability Resources (FHIR) is a more recent exchange standard that uses RESTFUL internet protocols and commands to facilitate exchange via prespecified templates, called Resources.^55^ The FHIR Specification recognizes a single attribute to represent all aspects of a patient’s gender, based on the fact that many systems and organizations only provide for a single attribute. This initial “must support” requirement is located in the FHIR patient resource element “patient.gender” and is coded with an internal FHIR Administrative Gender value set. This means that the work needed to disambiguate sex and gender data is left to implementers. Guidance has improved, with suggestions that are similar to those proposed in this document. For more information, see Section 8.1.7 of the FHIR Patient Resource.^56^

### Digital Imaging and Communications in Medicine

Digital Imaging and Communications in Medicine (DICOM) is the international standard that supports the exchange and processing of medical imaging information.^57^ The DICOM model was introduced in 1985, has been unchanged since 1995, and includes a single mandatory field to capture “Patient Sex” with allowed values of “Male,” “Female,” “Other,” and “Unknown.” This field is used to capture clinical sex, specifying a value in DICOM-compliant equipment for sex-linked characteristics. For example, “Patient Sex” is a variable used in Standard Update Value computations in nuclear medicine and used as a parameter into patient dose sensitivity models for radiation dose reporting. Statistical analysis reflecting the sex-linked characteristics of the studied populations is used as the basis of the computations and models. The inability to accurately represent the varying contexts of use (administrative sex, GI, and SFCU) in one field occasionally leads to inconsistency in the image results and reports created by DICOM equipment. Manual changes to patient’s SFCU fields can cause inconsistencies in downstream systems that use HL7 “administrative sex” or “administrative gender.”

### National Council for Prescription Drug Programs

National Council for Prescription Drug Programs (NCPDP) is an American National Standards Institute (ANSI)-accredited SDO representing the pharmacy services industry.^58^ NCPDP standards include a Gender field with “Female,” “Male,” “Unknown,” and “Non-binary” values. NCPDP recognizes the business need to document and communicate gender and sex assigned at birth separately. Doing so may have a positive impact on patient care, reducing issues related to obtaining medication without unnecessary delay in situations where gender and sex assigned at birth do not match for a patient. NCPDP is moving to include both Administrative Gender and Sex at Birth elements in its future state and is considering sunsetting the use of the value U (“Unknown”).

### X12

X12 is an ANSI-accredited SDO established in the early 1980s that develops and maintains standards for business-to-business Electronic Data Interchange.^59^ Currently, X12 transactions support the use of a single patient-level “Gender” element that conflates gender and sex. This element is expected to be used in all situations associated with the patient where it represents the “…Code indicating the sex of the individual…” with “Male,” “Female,” and “Unknown” allowed values. X12 is considering expanding allowable values to include “Nonbinary,” “Self-reported as Transgender,” “Not provided,” and “Unknown.” Further clarification is being considered to indicate that “Not Provided” should be used when gender cannot be sent due to reporting restrictions and “Unknown” should be used when gender is unknown.

### US Health IT Certification/United States Core Data for Interoperability

The United States Core Data for Interoperability (USCDI) version 1 was adopted by the Office of the National Coordinator for Health Information Technology (ONC) as a standard in its “Cures Act Final Rule.”^60^ The USCDI is a “standardized set of health data classes and constituent data elements for nationwide, interoperable health information exchange.” The USCDI represents the evolution of prior regulatory “data sets” (ie, Common Meaningful Use Data Set and Common Clinical Data Set) created as part of the ONC Health IT Certification Program for initiatives such as the Centers for Medicare & Medicaid Services Electronic Health Record Reporting Programs. USCDI version 1 includes a single element for sex named Birth Sex^61^ coded with HL7 V3 AdministrativeGender and NullFlavor. Although gender identity^62^ is not included in USCDI version 1, a health IT system must demonstrate that it can be captured to be certified using ONC’s “demographics” certification criterion, and gender identity is currently classified at Level 2 as part of the USCDI expansion process. In July 2021, the ONC published the USCDI version 2, which in addition to “sex (assigned at birth)” also references two new data elements “sexual orientation” and “gender identity” within the Patient Demographics data class. While the publication of USCDI version 2^63^ does not immediately require health IT systems to be able to transmit and receive these data, it does prompt work within the SDO community to ensure that such data are included in future versions of, for example, CDA and FHIR implementation guides. This in turn builds momentum within the health IT industry and a clearer, more predictable sense of ONC’s future regulatory proposals. Though not yet fully represented in the USCDI, the GHP’s work is now poised to help incrementally inform and reshape how sex and gender data are approached by administrative staff, informaticians, health professionals, software developers, and policymakers.

### Sex and gender terminology systems

Several terminology-focused SDOs are working to improve gender-oriented terminology. SNOMED International has authored concepts referenced by USCDI and some international models to describe gender-related topics. These concepts are almost always represented as clinical findings because they represent an interpreted assessment of likely discrete observations. In addition to Male and Female, the US Edition of SNOMED CT provides concepts related to transgender and nonconforming gender findings, although these concepts are also given transexual phrasing which is controversial. All of these concepts are currently identified as ways to represent gender identity in the ONC’s Interoperability Standards Advisory.^62^ Given the culturally specific approach to gender identity and recognition of sex-related characteristics, use of country-specific terms should be approached with caution internationally. Users should always be mindful of the context in which the encoded terminology is intended to be used, and that terms may require updating. Just because SNOMED CT has a concept “Male-to-female transsexual” does not mean that it is necessarily the best choice for gender-affirming design.

Regenstrief Institute Inc.^64^ continues to add LOINC^65^ concepts where needed to represent clinical observations that characterize the specific sex and gender observation of interest. Fortunately, the LOINC concepts representing these observations are less encumbered by culture-specific phrasing and may be used in most international settings. The gender harmony model was designed with the underlying intent to use LOINC concepts to represent the various model elements (ie, the question) and SNOMED CT^66^^,^^67^ concepts where possible for the resulting “answers.”

### It is time for change

There is a pressing requirement to routinely capture accurate and time-period bound expanded gender identity and sex observation clinical information to support safe, quality, and gender-affirming care for all people and to deliver accurate and timely clinical screening, diagnostic testing and interpretation, and other equity-promoting interventions for gender-marginalized people.^47^^,^^68^ Inconsistent approaches to the representation of sex and gender diversity in health information systems have led to the fragmentation of safe, quality, and efficient care for sex- and gender-marginalized people and have resulted in easily preventable harms such as breaches in patient privacy.^39^^,^^69–73^ As National LGBTQ Task Force Director Rea Carey notes:

It is outrageous that basic health care is being denied to transgender and gender non-conforming people and that so much additional trauma is being caused by doctors instead of being resolved by doctors. The medical profession must take these data seriously and ensure that everyone in the medical care system knows how to provide transgender-sensitive medical care.^34^

## HL7 GENDER HARMONY PROJECT

The GHP was born in May of 2019 from the ongoing frustrations that sex and gender concepts are not accurately captured within existing health models and standards, impacting the quality of care for sex- and gender-marginalized people and other people, resulting in health inequities.^40^ It is a collective, collaborative, international effort to help fulfill health care’s responsibility to gender-marginalized people by specifying gender-inclusive standards that can be used by systems and clinicians in the provision of affirmative and quality person-centered care. The primary output of the GHP to date is the HL7 Gender Harmony Logical Model.

### HL7 standard development process

The Model has been developed as part of an ongoing effort within the HL7 GHP by following the ANSI-conformant standards processes,^74^ which require open meetings, documented participation, and procedural observance of Robert’s Rules of Order.^75^ The community that produced the Model met weekly over the course of 2 years. The GHP community is transdisciplinary and includes participants representing the interests of gender-marginalized people with lived experience, international implementers such as electronic health record vendors and clinical application experts, academics, standards bodies, government agencies, and HL7 subject matter experts.^12^ The GHP used a consensus-based, expert-driven approach to specify the data elements, value sets, element attributes, and use cases, which have been integrated into a harmonious logical model outlined in an HL7 informative ballot. This informative document provides guidance but does not specify conformance requirements.^74^ An informative document ballot type was chosen by the project because of the transformative nature of the Model content and the variety of data representation found in current models and implementations. At the inception of GHP, a uniform approach that could be adopted in a consistent manner across the various existing models had yet to emerge. In January 2021, the GHP submitted the informative ballot for review, comment, and voting.^13^ Reviewers of the ballot provided over 200 comments and the diverse members of the gender harmony community openly debated and resolved all comments. All resolutions for “Negative” comments were either directly approved by the original commenter or were made available to them for acceptance. Resolution of comments and edits resulted in the Gender Harmony publication made available by HL7 as a final publication of the specification.^41^

## HL7 GENDER HARMONY LOGICAL MODEL

**Figure 1.**
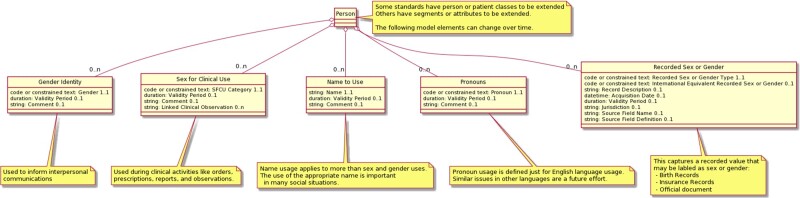
HL7 Gender Harmony Logical Model describes information model elements using Unified Modeling Language (UML) type diagramming that illustrate how information models, particularly exchange models but also data representation models, could characterize patient gender and sex information.

The Model is a conceptual model that outlines the data elements, values sets, element attributes, and relationships that clarify the meaning and context of the information presented to guide and inform changes within operational standards. The Model has 5 major elements independent of other components that may also be part of the information model for a person: Gender Identity, Sex For Clinical Use, Recorded Sex or Gender (RSG), Name to Use (NtU), and Pronouns . A general Unified Modeling Language^76^ diagram of the Model is presented in Figure 1.

### Gender harmony logical model elements

The Model is informed by, but not restricted to, existing HL7 standards-based models and directly addresses the challenges as outlined. Unreliable sex and gender data captured for administrative and legal uses can be appropriately distinguished from clinical concepts by noting the item is a specific recorded sex or recorded gender. Limitations imposed by the binary representation of sex and gender are addressed by enabling expanded sex and gender codes in value sets. The Model introduces elements that enable affirmative care by providing patient-specified names and pronouns (by identifying NtU and Pronouns). The elements of the Model support affirming clinical interactions with all patients, and reliable information to clinicians. Possible values for elements in the Model are provided in Supplementary Appendix S1.

- *Gender Identity*: GI is an individual's personal sense of being a man, woman, boy, girl, or something else. This element is proposed to have a minimum list of values (see Supplementary Appendix S1) that may be extended with additional values suitable for the local context. This model explicitly states that GI is something that is determined by a person themselves and cannot be assigned. Therefore, an infant or other person that cannot express their preferred GI cannot have a GI, yet they may have sex observations (see SCFU below) and may have a sex assigned at birth (which would be noted as a recorded sex).
- *Recorded Sex or Gender*: The RSG element is used to more accurately identify sex values or gender values that are specified in a particular source or documents such as identity cards or insurance cards. By characterizing these sex or gender data found or obtained for a specific use as “recorded” along with documenting the context of use, systems avoid repurposing administrative sex and gender data inappropriately (ie, for clinical care). RSG can be used to capture specific gender records used for administrative purposes or to provide background demographic information about the patient. A single recorded administrative sex or gender value is often inappropriately assumed to be all that is needed to understand the patient's clinical sex and gender identity. We propose that when the intended meaning of the RSG datum is unknown, identifying the document type or context that a sex or gender value was recorded within can help reduce unintended assumptions on how the value should be applied.
- *Sex For Clinical Use*: SFCU is a summary sex classification element based on one or more clinical observations such as an organ survey, hormone levels, and chromosomal analysis. SFCU can provide a “patient-level” summary clinical sex characterization value to be specified for any clinical order, result, or assessment. SFCU also allows users to specify different values for the same patient for specific clinical uses. For example, SFCU can be used to justify instrument set-up based upon an organ inventory observation or hormonal levels. The SFCU element provides the option to refer to specific clinical observations or reports to clarify the value selection. Specification of values for this element allows for assumption-free clinical care, facilitating references to reports, organ inventories, or other artifacts essential to quality care. As noted in Supplementary Appendix S1, allowed values for SFCU include Male, Female, and Specified. The SFCU value “Specified” is preferable to the term “Other” that is found in many value sets because it is nonstigmatizing and explicit. In addition, based on discussions during model development, including discussions with members of the intersex community, the phrase “Intersex” is not included in the allowed values because the phrase is specific, overly revealing, and can be over-interpreted. While it may be true that at a general level an intersex individual would be represented as SFCU = Specified, a specific SFCU value for a particular test could be contextually narrowed to male or female. SFCU is engineered to address real-world systems and situations that can reflect gender and sex characteristic alignment, and also flag situations where a collection of observations does not fall into a binary value or is otherwise specified by the user. The list of allowed values for this element is listed in Supplementary Appendix S1.
- *Name to Use*: The NtU element identifies the name that the patient has indicated they wish to use in healthcare interactions. This element may match but is distinct from a person’s legal name and is the appropriate name to be used in person-centered healthcare conversations. This element will have benefits beyond those of gender-inclusive care: people with Americanized names, people with very long names, and people with preferred names will be able to inform clinicians of those names without having to change their legal name.
- *Pronouns*: The pronouns element identifies the English language third-person personal pronoun set used by the patient. A pronoun set is defined as a set of personal pronouns (subject and object pronouns) and their respective possessive pronoun(s), reflexive pronoun(s), and possessive determiner(s) (colloquially referred to as “possessive adjectives”). These values are specified by the patient for use in healthcare interactions, clinical notes, and in written instructions to caregivers (Supplementary Appendix S1). Based on input during balloting the model, a specific international attribute was added to support accurate mapping across different character sets. Implications of pronoun use in non-English languages are not addressed by the Model.

### Model-element attributes

Each of the sex and gender elements described above also have the following characteristics and optional associated attributes.

The model, as represented in the Unified Modeling Language structure in Figure 1, notes that each of the 5 model elements may *not* occur in a patient record, or may occur many times. This is represented using the “0..n” notation. Given that each model element includes a *Validity period*, a patient record is allowed to have a series of the same element that use the validity period to clarify when that element is to be considered “active” and as such, an implementation may support multiple of the same element, such as GI, at the same time. We would expect that when this is allowed to occur the Comment attribute (or similar) would clarify the different context that any concurrent value should apply to. Attributes with a “1..1” notation are required and may only exist once for that specific record. We use this for each of the attribute values that carry the primary information for that element, such as the *Gender* attribute in the *GI* element. Most attributes within each element use the “0..1” notation which indicates that the attribute is optional (may not exist or exist only once per instance of data).

- *Validity* *period*: when present, indicates the time period that the grouped information for the data element is valid. When no “end date” is provided, it can be used to indicate the date and time that a report was written or a specific date/time an observation was made. For a document like an identity card, this information should be information obtained directly from the card.
- *Comment*: a general-purpose attribute that provides additional contextual information for the data element. In this simple Model, the comment attribute is a malleable data attribute that when integrated into a specific implementable model (such as FHIR) will be transformed into one or more model-specific attributes. When included in an SFCU, it can describe the intended clinical context that the SFCU value is intended to be used within. For an RSG, the comment may provide additional complexities about the record, for an NtU, this may identify the clinical situation this name is to be used in.

### Adoption considerations

The Model is intentionally abstract. Data elements and attributes need to be mapped to the data classes specified in existing standards such as FHIR and DICOM to be adopted. Existing standards have patient information models that provide some, but not all, of these data elements. There are currently efforts underway to update these standards to align with this work including:

- Mapping between the information models where both provide equivalent elements.
- Adding elements where the existing standard lacks an equivalent element.
- Reconciling and partially mapping similar (but not equivalent) elements between this model and the standards provide elements.

## DISCUSSION

A small number of expert healthcare organizations have been collecting sex and gender data in clinical systems for years^69^; however, restrictive “context-framing” such as the use of “Administrative gender” has oversimplified the complex and dynamic nature of sex and gender and led to inconsistent approaches to the representation of these data in systems. Consistency is required for interoperability but has been hampered by a lack of a standard model that can be widely adopted and that outlines common definitions, structure, and terminology. Despite this barrier, some clinical systems vendors and health organizations have independently crafted system-specific changes that support better sex and gender data collection.^47^ While admirable, a standard model for the creation of data and exchange standards defined within SDO specifications is required for consistency and wide adoption. Relying on market innovation to drive widespread harmonized adoption is insufficient. Software developers may still need incentives to adopt model standards through policy, program, and/or regulatory changes. This process will require iterative improvements and ongoing maintenance. The healthcare community must be engaged and open to incremental improvement.

To develop this model the HL7 project worked hard to reach out to all members of the impacted community and when active participation could not occur on a weekly basis, material was circulated to gender-marginalized communities and others. Our approach to intersex persons follows the guidance from intersex advocacy group, interACT, where they state:^77^

While some people with intersex variations do hold their intersex status as an important part of their gender, intersex should not be added as a gender identity option. We find that including the term as an answer to a question on gender identity risks drawing false positives—people without intersex traits who may think the word is synonymous with gender-related terms like nonbinary or transgender.

It will be important for SDOs to stay informed about the needs of gender-marginalized people and to engage with the community to best understand how to improve. LOINC is seeking input into any new requests that are needed to align with finalized sex and gender observations and data elements. The NCPDP has begun incremental improvements in SCRIPT ERx to support sex and gender representation where necessary.^78^ DICOM has been participating in the GHP and has an active change proposal, CP1927, for modifications to extend DICOM to be consistent with the Model. Health terminology standards are essential to the exchange of accurate and meaningful sex and gender information in a manner consistent with the Model.

The HL7 community of standards has begun to work with the members of the current GHP to incorporate the proposed changes into each of the existing HL7 standards—V2, CDA, and FHIR. The improvements will be based upon a common framework resulting from the published Model. Work has started on applying the Model to the US Core^79^ FHIR specification, and discussions with the groups responsible for the FHIR Patient resource have been initiated. Alignment has already begun based on immediate needs driven by COVID-19 data capture and analysis. Given the rush to move forward, other SDO work may happen in parallel. Members of the community are committed to maintaining alignment. The desired outcomes of these parallel efforts include consistency, completeness, and accuracy of data definition, collection, exchange, and use. These improvements will enable quality health care and improved outcomes for gender-marginalized people. Preventable harms due to absent, inaccurate, or conflicting access to information can also be reduced. The incorporation of expanded sex and gender data in clinical decision support tools and algorithms should enable clinicians to accurately document clinical findings and provide service offerings based on measurable data (eg, improved radiation dose modeling). To advance the goal of equity in access to quality care, additional training, and education for healthcare providers is a critical, complementary requirement.^36^^,^^37^^,^^40^^,^^47^^,^^80^ An educated community of care providers enabled with an improved technical framework that accurately represents diverse sex and gender data will indeed enable better health outcomes.

When these improvements are implemented based on standards accompanied by certification expectations, exchange of these data between healthcare organizations will improve the patient experience by reducing requirements for data re-entry and improving the reliability of sex and gender information made available to clinicians, enabling quality care relationships for gender-marginalized people from intake.

## CONCLUSION

Most clinical systems and current standards in health care do not consistently or fairly represent sex and gender diversity. This has impeded interoperability, constrained information that supports safe and affirming care, and led to preventable harms for sex- and gender-marginalized people. The Gender Harmony Project was formed to specify requirements for inclusive health information exchange standards and develop a model that enables health equity. The Gender Harmony Model provides informative guidance for standards developers to implement a more thorough technical design and improves upon the binary design used in many legacy clinical systems.

## FUNDING

This research received no specific grant from any funding agency in the public, commercial or not-for-profit sectors and represents the work of an international collaboration of volunteers and in-kind contribution by those participating in HL7 International.

## AUTHOR CONTRIBUTIONS

RCM conceived the research idea and is the primary author of the manuscript. RCM, CLM, and KD contributed several critical revisions and provided significant feedback on discussed systems. RCM provided oversight and input throughout the document. CLM, RQ, and SP are the primary authors of the current state section. RJH, RCM, and CLM are the primary authors of the model section. All authors participated in writing, and CG, RCM, and KD are the primary authors of the discussion section. CK is the primary author of the background section and made critical contributions to affirmative language and content throughout. KD contributed to the background, problem, conclusion, and other sections. All authors participated in the development of the appendices. All authors participated in revisions and have reviewed the final manuscript for accuracy and completeness. Authors agree to be accountable for all aspects of the work in ensuring that questions related to the accuracy or integrity of any part of the work are appropriately investigated and resolved.

## SUPPLEMENTARY MATERIAL

Supplementary material is available at *Journal of the American Medical Informatics Association* online.

## Supplementary Material

ocab196_Supplementary_Appendix
