# Corrigendum to: Gender harmony: improved standards to support affirmative care of gender-marginalized people through inclusive gender and sex representation

**DOI:** 10.1093/jamia/ocab255

**Published:** 2021-11-25

**Authors:** Robert C McClure, Caroline L Macumber, Clair Kronk, Chris Grasso, Robert J Horn, Roz Queen, Steven Posnack, Kelly Davison

*Journal of the American Medical Informatics Association*, ocab196, https://doi.org/10.1093/jamia/ocab196

In the originally published version of this manuscript, there were several errors, which are listed in this corrigendum as follows:

The acronym “SCFU” in the sections “Sex and gender in clinical systems”, “Health Level 7 Version 2”, “Digital Imaging and Communications in Medicine” and “Gender harmony logical model elements” should read “SFCU”.

The acronym “2SLGBTQIA+” which appears in the “Background” section has been added to “Table 1: Glossary of additional key terms” with the following definition “acronym meaning two-spirit, lesbian, gay, bisexual, transgender, queer, intersexual, asexual, while “+” indicates that some individuals may express gender and sexuality in others ways”.

These have been corrected online.

