# Supplementary material for: Gender harmony: improved standards to support affirmative care of gender-marginalized people through inclusive gender and sex representation
Source: J Am Med Inform Assoc. 2021 Oct 6;29(2):354–63. doi: 10.1093/jamia/ocab196 (PMC8757317; doi:10.1093/jamia/ocab196)
Supplement: ocab196_Supplementary_Appendix [file ocab196_supplementary_appendix.docx]

**APPENDIX 1**

**BASELINE VALUE SETS**

The material in the appendix is drawn directly from the HL7® Informative Document: Gender Harmony - Modeling Sex and Gender Representation, Release 1 specification[76].

**Gender Identity**

The proposed baseline value set contains four categories that are intended to be the minimum exchanged values. This is expected to be extended with locally appropriate additional terms. Additional values should be mapped to one of the four minimal values to support minimal interoperability. By utilizing minimum value sets, we are allowing individual countries and regions to use terminology that they themselves find most useful, i.e. terminology in some regions does not necessarily exist to the same degree in others. For instance, Two-Spirit makes sense to include in a North American context, but may not have traction or use at all, in say, Saudi Arabia. We focused our minimum value sets on translatability and penetration into linguistic subgroups on an international scale. Examples of gender identity extensions can be found at http://hl7.me/GHP. The full list at a specific locality can be much larger and more detailed.

| **Term** | **Definition** |
| --- | --- |
| Female | A person's self-identification as a woman, as female, or as a girl. |
| Male | A person's self-identification as a man, as male, or as a boy. |
| Nonbinary | Having a specific identity which is not within a binary construct of male or female or having an identity which falls under the umbrella of any or all gender identities which are not female or male. |
| Unknown | The person’s gender identity is not known at this time, for any of a variety of reasons.  (E.g., “was not able to ask” or “person does not want to answer”.) |

**Recorded Sex and Gender**

There is no baseline set of expected values for recorded sex data or recorded gender data because the intent is to faithfully represent the text found in the source material. There are many abbreviations and codes found on current and historical documents. The RSG element includes an attribute to identify the source document so that further information for that kind of document or record can be found.

**Sex for Clinical Use**

| **Term** | **Definition** |
| --- | --- |
| Female | The “female” values apply to this patient, in the case of a given procedure or process in a given context. |
| Male | The “male” values apply to this patient, in the case of a given procedure or process in a given context. |
| Specified | This patient has specific documented characteristics that do not fully match either male or female in a given context. |
| Unknown | There are insufficient observations to determine a meaningful SFCU.  For example, an emergency trauma case may require treatment before SFCU can be established. |

This baseline set of proposed values for exchange is intended to be a categorization of one or more sex-related observations that are to be utilized within a specified context. Use of the set should be restricted so that only values in the set are allowed. The concept “Specified” is a flag to indicate non-standard sex observations. This term “Specified” was chosen because it’s meaning aligns with the intended use to flag that a specified sex categorization in the associated context requires review of additional clinical artifacts, such as physiologic sex observations. Those observations can be linked directly to the exchanged value, but such linkages are not required. This term was chosen after long deliberation because other terms under consideration, such as “Other” and “Complex” may be considered both ambiguous and stigmatizing. Use of SFCU “Specified” allows users to indicate neither “Male” or “Female” is correct and then reference specific observations thereby reducing the need for users to make clinical assumptions and facilitating objective clinical investigations, screening, treatment and referral decisions.

**Pronouns**

The baseline value set for pronouns corresponds to the LOINC® Answer List for Personal Pronoun Codes. The answers reflect the subjective/objective/dependent possessive/independent possessive/reflective cases in English grammar.

| **LOINC Observation: 90778-2 Personal Pronoun Codes**  **LOINC Answer List: LL5144-2 Personal pronouns / Answers: 10; Scale: Nom; Code: -; Score: -** | |
| --- | --- |
| **Answer** | **ID** |
| **he/him/his/his/himself** | **LA29518-0** |
| **she/her/her/hers/herself** | **LA29519-8** |
| **they/them/their/theirs/themselves** | **LA29520-6** |
| **ze/zir/zir/zirs/zirself** | **LA29523-0** |
| **xie/hir ("here")/hir/hirs/hirself** | **LA29521-4** |
| **co/co/cos/cos/coself** | **LA29515-6** |
| **en/en/ens/ens/enself** | **LA29516-4** |
| **ey/em/eir/eirs/emself** | **LA29517-2** |
| **yo/yo/yos/yos/yoself** | **LA29522-2** |
| **ve/vis/ver/ver/verself** | **LA29524-8** |

**APPENDIX 2**

| Acronyms | | | | |
| --- | --- | --- | --- | --- |
| ADT | Admit Transfer Discharge |  | HL7 V3®/V3 | Health Level Seven Version 3 |
| ANSI | American National Standards Institute |  | LOINC® | Logical Observation Identifiers Names and Codes |
| DICOM | Digital Imaging and Communications in Medicine |  | NCPDP | National Council for Prescription Drug Programs |
| FHIR® | HL7 Fast Healthcare Interoperability Resources |  | NtU | Name to Use |
| GHP | Gender Harmony Project |  | PID-8 | Patient Identifier, Segment 8 in the HL7 V2 specification |
| GHM | Gender Harmony Model  (“the Model”) |  | SDO | Standards Development Organization |
| GI | Gender Identity |  | SFCU | Sex for Clinical Use |
| HL7 C-CDA®/ CDA | HL7 Consolidated Clinical Document Architecture |  | RSG | Recorded Sex or Gender |
| HL7 | Health Level Seven International® |  | USCDI | United States Core Data for Interoperability. Initially published as version 1 (V1) and in 2021, published as version 2 (V2) |
| HL7 V2®/V2 | Health Level Seven Version 2 |  |  |  |
